# Evaluating human papillomavirus (HPV) self‐sampling among Latinas in the United States: A systematic review

**DOI:** 10.1002/cam4.70098

**Published:** 2024-08-16

**Authors:** Marisol S. Cora‐Cruz, Omar Martinez, Sophia Perez, Carolyn Y. Fang

**Affiliations:** ^1^ Cancer Prevention and Control Program Fox Chase Cancer Center Philadelphia Pennsylvania USA; ^2^ College of Medicine University of Central Florida Orlando Florida USA

**Keywords:** cervical cancer, human papillomavirus (HPV), Latinas, screening, self‐sampling

## Abstract

**Background:**

Latinas experience the greatest cervical cancer incidence compared with other ethnic/racial groups in the United States (US) due in part to significant disparities in screening uptake. Social and structural conditions that impede access to and participation in screening include language barriers, concerns about documentation status, logistical issues (e.g., transportation, limited clinic hours), and cultural beliefs regarding modesty and promiscuity. To overcome these challenges, self‐sampling for human papillomavirus (HPV) DNA testing has emerged as a potentially promising method for promoting cervical cancer screening among this population. Thus, this systematic review aimed to assess the acceptability of HPV self‐sampling among US Latinas.

**Methods:**

Using EBSCOhost and PubMed databases, we searched for studies published in the past two decades (2003–2023) that described participation in HPV self‐sampling among Latinas. Eleven articles met inclusion criteria.

**Results:**

The majority of studies were conducted in Florida, California, and Puerto Rico, were single‐arm designs, and involved the use of community health workers and Spanish‐language materials (e.g., brochures). Across studies, the majority of participants reported that self‐sampling was acceptable with respect to ease of use, comfort (lack of pain), privacy, and convenience; however, some women were concerned about the accuracy of self‐sampling or whether they had performed sample collection correctly.

**Conclusion:**

Given the high acceptability, self‐collection of cervicovaginal samples for HPV testing may offer a feasible option for enhancing participation in cervical cancer screening in this population that encounters significant barriers to screening.

## Abstract 2

This systematic review summarizes the acceptability of HPV self‐sampling for cervical cancer screening among US Latinas. Studies found that self‐sampling was acceptable due to its ease of use, comfort, privacy and convenience. Therefore, it presents a viable option for enhancing participation in cervical cancer screening in this population.

## 1 INTRODUCTION

Due to advances in the early detection and prevention of cervical cancer, deaths from cervical cancer are declining. However, not all groups are benefitting equally from these advances. Notably, cervical cancer incidence remains 32% higher among Latina women in the continental United States (US) and 78% higher among women in Puerto Rico (a US territory) compared with non‐Hispanic White (NHW) women.^1^ National statistics report that cervical cancer incidence rates are 9.3/100,000 among Latinas compared to 6.7/100,000 among NHW women. Although cervical cancer mortality rates have declined by ~35% from 1990 to 2019 across both groups, Latinas still experience higher death rates and are 30% more likely to die from cervical cancer than NHW women (2.0/100,000).^1^

Cervical cancer is a highly preventable disease, largely due to effective screening tests and the development of a vaccine against high‐risk human papillomavirus (HPV). The US Preventive Services Task Force (USPSTF) recommends routine cervical cancer screening for women between the ages of 21 and 65.^2^ Yet, despite having one of the highest incidence rates of cervical cancer, Latinas are significantly less likely to participate in cervical cancer screening compared with NH White and Black women.^3^ As of 2021, 67.9% of Hispanic women, compared to 75.7% of NHW women, reported being up to date with current cervical cancer screening guidelines.^4^ Furthermore, only 87% of Hispanic women report ever having had a pap smear, compared to 94% of NHW women.^5^ Previously identified barriers to screening include: language barriers, lack of access to care or limited clinic hours, racism and discrimination, inadequate knowledge, preference for race/ethnic concordant providers, and cultural concerns regarding modesty.^6^, ^7^ Peer approaches and community‐driven interventions may be successful in establishing trust and providing education about screening,^8^ but structural barriers (e.g., inconvenience of clinic hours) and limited access remain formidable challenges to overcome and are not easily addressed with traditional health promotion programs.

Emerging technologies supporting self‐sampling for human papillomavirus (HPV) testing have generated considerable interest^9^, ^10^, ^11^ in light of data that self‐sampling (i.e., the use of self‐collected cervico‐vaginal samples) for HPV DNA testing yields comparable accuracy with that of HPV‐testing performed on clinician‐obtained samples.^12^, ^13^, ^14^, ^15^, ^16^ Further, studies have demonstrated that self‐sampling for HPV testing is an effective approach for increasing screening coverage among non‐compliant women in other populations and countries.^17^, ^18^, ^19^, ^20^, ^21^ Convenience of self‐sampling is reported to be a key advantage of self‐sampling,^22^ particularly among those who report that it is difficult to obtain clinic‐based screening due to a lack of time or inflexible clinic hours.^23^

Simply expanding access to these tools, however, may be insufficient to promote participation and interest in screening. In this regard, community health workers (CHWs) have emerged as assets in promoting cancer prevention and screening, particularly among marginalized communities.^24^, ^25^, ^26^ Often hailing from historically underrepresented groups, CHWs not only bring valuable cultural and linguistic insights that foster trust and rapport within their communities, but also are trained to bridge gaps in access to care and empower individuals to make informed decisions about their health.^27^ In fact, a recent study found that CHW‐facilitated self‐sampling may be useful for mitigating multilevel barriers to cervical cancer screening among Latina women.^28^ As a result, the integration of CHWs with self‐sampling options may enhance the acceptability and efficacy of this strategy for promoting cervical cancer screening.

National guidelines now include primary HPV screening every 5 years as an evidence‐based cervical cancer screening strategy for women ages 30–65, and it was given an “A” recommendation by the USPSTF.^2^ Because self‐sampling for HPV testing in non‐clinical settings has comparable accuracy to HPV‐testing on clinician‐obtained samples,^12^, ^13^, ^14^, ^15^, ^16^ the USPSTF suggests that “…self‐collection may be one strategy for increasing screening rates among populations where they are currently low.”^2^ Hence, HPV self‐sampling may offer a feasible and efficient approach for significantly increasing screening coverage among Latinas, a population that has traditionally had lower cervical cancer screening rates than other groups. However, the acceptability of this approach among Latina women in the United States has not been systematically evaluated. Thus, the present paper aims to review published studies that examined self‐sampling approaches for cervical cancer screening in US Latina women.

## 2 MATERIALS AND METHODS

We conducted this systematic review according to the Preferred Reporting Items for Systematic Review and Meta‐Analyses (PRISMA) guidelines,^29^ which ensures the highest standard in systematic reviewing. The articles were identified by the authors and the librarians at the University of Central Florida College of Medicine and were evaluated independently, as described below.

### 2.1 Search strategy

Four sets of search strings were built based on the following concepts: (1) cervical cancer screening, (2) self‐sampling, (3) Hispanic community, and (4) programs or interventions. The publication year filter was used to narrow down studies between 2003 and 2023. This date range was selected because HPV DNA testing (combined with a Pap test) was first included in US screening guidelines starting in 2003.^30^ The search was conducted using EBSCOhost (which contains 91 databases) and PubMed. In addition to the authors, the search was supported by two University of Central Florida College of Medicine librarians. Upon their initial search, the librarians identified a dearth of clinical trials focused on HPV self‐sampling among underserved populations, particularly Latinas. Thus, the librarians helped to expand the inclusion and exclusion criteria search terms for the literature search. See Table S1 for the comprehensive list of search terms used.

### 2.2 Inclusion criteria

To broadly identify research on the acceptability of self‐sampling in the context of cervical cancer screening among US Latinas, studies were included if they: (1) were published between January 2003 and August 2023, (2) were conducted in the US, (3) included self‐collection of a cervical or vaginal sample, (4) enrolled participants who self‐identified as Hispanic or Latina, (5) reported acceptability or uptake of self‐sampling, and (6) were published in English or Spanish. Articles were excluded if they: (1) focused exclusively on the concordance of HPV DNA test results between self‐collected and clinician‐collected samples; (2) were review papers, commentaries, or protocol/methods papers; or (3) did not include a study sample that was comprised of at least 10% of participants who self‐identified as Hispanic or Latina.

### 2.3 Screening process and data extraction

The review procedure included the following steps: (1) First, two reviewers independently screened each paper title and abstract for eligibility. (2) Relevant articles were then selected for full‐length review. (3) Studies included in the full‐length review underwent data extraction, with one reviewer completing the extraction of key study elements and the other reviewer appraising for accuracy. Disagreements at each stage were discussed among all authors until consensus was achieved. Key study elements within the standardized extraction template included the location of the study (where the study was conducted), study purpose, sample size (including the proportion of Latina participants), mean participant age, self‐sampling device(s) used, description of the study procedure(s) or intervention(s), study length, any cultural components, and main acceptability and uptake outcomes.

## 3 RESULTS

**FIGURE 1:**
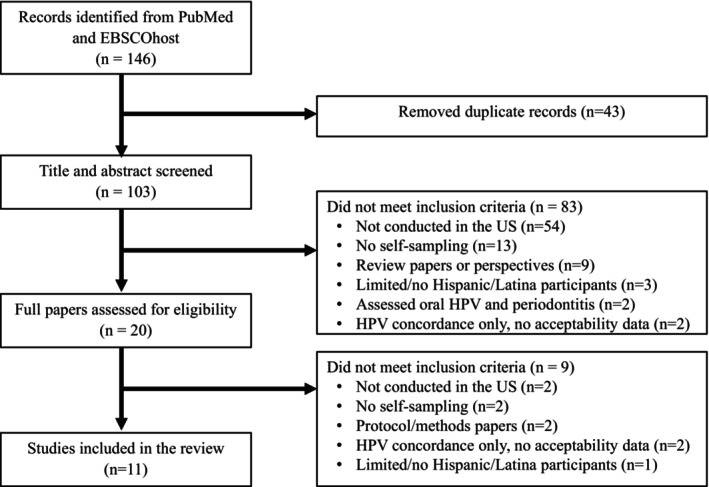
Preferred Reporting Items for Systematic Reviews and Meta‐Analyses (PRISMA) flow diagram of search results.

The database search yielded 146 citations. After removing duplicates, the remaining 103 studies were subjected to title and abstract review. Twenty studies were assessed at full length, of which 11 were included in the systematic review. See Figure 1 for Flow Diagram.

### 3.1 Study characteristics

Sample sizes ranged from 100 to 1213 participants; all studies except one^31^ had 601 or fewer participants. Two studies were published between 2005 and 2008, five between 2012 and 2016, and four between 2018 and 2023. No studies published between 2009 and 2011 met the inclusion criteria. Multiple studies were conducted in southern Florida (*n* = 3), California (*n* = 2), and Puerto Rico (*n* = 2). Other study locations included New York, North Carolina, Texas, and the U.S.‐Mexico Border (state not specified). Two studies were randomized control trials^32^, ^33^ and the remainder were cross‐sectional or single‐arm studies.^31^, ^34^, ^35^, ^36^, ^37^, ^38^, ^39^, ^40^, ^41^ See Table 1 for additional description of the Study Characteristics.

**TABLE 1:** Characteristics of studies included in the review.

Author, (year)	Location	Purpose	Sample size	Latino sample (% of total)	Sampling method or device	Description	Length	Cultural components	Main outcomes
Intervention studies with only Latina/Hispanic participants									
De Alba et al., (2008)^31^	Orange County, California	Assess the sensitivity/specificity of self‐sampling and satisfaction with collection methods	1213 completed self‐sampling; 622 also had a clinic visit to obtain a Pap test and the first 386 of these had a clinician‐collected sample for HPV testing Age range: 18 years or older	100% 89.4% Mexican/Mexican American, 6.5% Central American, 3.5% South American, 0.6% other Hispanic (not specified)	Vaginal self‐sampling kit (not specified further)	Lay health workers provided self‐collection kits to women in community settings and instructed them on use. Self‐collection was performed at the participant's home or other convenient location. Samples were collected by lay health workers and processed within a week after self‐collection	Not specified	Use of Spanish‐speaking lay health workers for instructing the participants on how to self‐collect a sample. Written instructions for self‐collection were provided in Spanish	64.5% reported having an excellent (33.7%) or very good (30.8%) experience with self‐sampling 81.8% rated convenience of self‐sampling over clinician sampling at clinic as excellent or very good
Montealegre et al., (2015)^38^	Harris County, Texas	Assess acceptability and feasibility of self‐sampling in a sample of predominantly Mexican immigrant women	100 Age range: 21 years or older	100% 98% immigrant Mexican women and 2% Salvadorean	Cytology broom (Hologic), vial with liquid‐based cytology fixative (Thin Prep®, Hologic)	Participants were provided with an instructional brochure on how to complete self‐sampling. Self‐collection occurred at the Mexican consulate in Houston, which offers on‐site health services	1 time point	Brochure was in the participant's native language	Post‐sampling, 83% agreed the device was easy to use, and 98% agreed the instructions were easy to understand; 91% were “very willing” to use self‐sampling regularly. Among the 94 women who had prior Pap testing, 69.2% believed self‐sampling was more convenient compared to Pap tests
Non‐randomized intervention studies with mixed samples									
Ortiz et al., (2012)^40^	University of Puerto Rico, Medical Sciences Campus, Puerto Rico	Assess acceptability of cervical and anal self‐sampling	100 Age range: 18–34 years	94% 87% were born in Puerto Rico, 7% born in the Dominican Republic	Cytobrush (for cervicovaginal specimen) and Dacron Swab (for anal specimen)	Clinician performed a pelvic exam and collected cervical and anal samples. Participants were then provided verbal and written instructions on how to self‐collect cervical and anal samples. Self‐collection was completed at the clinic	1 time point	Survey items were drawn from an instrument developed for screening in a Mexican population	50% found cervical self‐collection to be more acceptable than clinician‐collected methods, and 28% did not have a preference between methods^b^ 43% found anal self‐sampling more acceptable than clinician‐collected sampling, and 35% found it equally acceptable^b^ 71% would like to complete self‐sampling if the kit was mailed to their home^b^
Ortiz et al., (2015)^41^	San Juan Metropolitan area, Puerto Rico	Assess acceptability and feasibility of cervical, anal, and oral self‐sampling	566 consented; 564 provided samples Age range: 16–64 years	88.7% The 88.7% were born in Puerto Rico	Dacron Swab (vaginal and anal specimens) and mouthwash (oral sample)	Study staff visited participants at home and provided verbal and written instructions for self‐collection of anal, vaginal, and oral samples. Sample collection was completed at the participant's home Participants were asked their preference for clinician‐ or self‐sampling for each type of sample	1 time point	Interview instruments were previously piloted within the community. Self‐collection instructions were provided in Spanish	Comfort with self‐collection method highest for oral (89.9%), followed by cervical (57.4%) and anal (49.2%)^b^ Participants preferred self‐sampling over clinician sampling for cervical (64.6%), anal (71.9%), and oral (65.7%) samples^b^ 89% were willing to complete self‐sampling if kit was sent via mail^b^
Penaranda et al., (2015)^35^	U.S.‐Mexico Border	Assess acceptability of vaginal self‐sampling among women in the U.S.‐Mexico border	110 Age range: 30–65 years	87% 50% were born in Mexico	Cervicovaginal self‐sampling kit (not specified further)	Participants received a 30–45‐min educational session with a bilingual *promotora* along with instructions on self‐sampling. Self‐sampling was completed in a clinic bathroom	1 time point	The intervention was delivered by a bilingual *promotora*. Information provided came from previous focus groups with this population	Acceptability was greater for self‐sampling (25.0, SD: 2.9) than for Pap test (22.7, SD: 3.0)^b^. All participants (100%) reported that self‐sampling was easy and convenient^b^ 29% of participants believed Pap tests were painful, as opposed to 15% reporting that self‐sampling was painful^b^ 30% of participants reported a preference for self‐sampling, 26% reported a preference for the Pap test, and 42.7% expressed no preference^b^
Anhang et al., (2005)^34^	New York City, New York	Assess acceptability of self‐sampling compared to clinician‐collected sampling among low‐income minority women	287 172 (60%) also completed the study questionnaire Age range: 25–65 years	76.50% this included 39.2% from Dominican Republic, 6.6% Colombian, 5.4% Mexican, 4.8% Puerto Rican	Cervicovaginal self‐sampling using Dacron Swab	Self‐collection was completed in a private restroom within the clinic. Afterward, a healthcare provider conducted a pelvic examination and obtained a cervicovaginal sample	1 time point	Survey was provided in English and Spanish	28.3% of Latina participants^a^ preferred self‐collection over the clinician‐collected procedure Overall, participants^b^ reported that self‐sampling was easy to use (69%), not painful (62%), and private (52%). However, 31% of participants^b^ were unsure whether they correctly collected their samples
Ilangovan et al., (2016)^37^	Miami‐Dade County, Florida	Assess acceptability and feasibility of self‐sampling	180 Age range: 30–65 years	74% Of these, 43% were Cuban, 12% Nicaraguan, 11% Honduran, and 10% Colombian. The remainder was from 10 other Latin American countries	Cervical‐vaginal sampling using the POI/NIH self‐sampler	Participants were recruited from the waiting room of a safety‐net clinic and received a brief educational session on cervical cancer screening delivered by a CHW, including information about Pap tests and an explanation of HPV self‐sampling. Women were asked to select either self‐sampling on‐site before provider visit or to discuss the need for a Pap test with their clinician. Self‐sampling was completed at the clinic	1 time point	Educational materials were tailored to the community	60% of Latinas^a^ opted for self‐sampling over the Pap test 99% of Latinas^a^ stated the instrument was easy to use, 96% felt they collected their sample correctly, and 98% would recommend self‐sampling to family and friends^a^. 16% experienced discomfort while obtaining their sample^a^. 89% would choose self‐sampling over a Pap test in the future^a^
Rohner et al., (2020)^36^	University of North Carolina Women's Hospital and Duke University Hospital, North Carolina	Assess acceptability of urine self‐collection and cervico‐vaginal self‐sampling	410 Age range: 25–65 years	29%	Cervico‐vaginal sample collected using Viba Brush (Rovers Medical Devices BV, The Netherlands)	Participants received verbal and written instructions for self‐sampling. They collected two urine samples and completed cervico‐vaginal self‐sampling at a clinic. Participants also received care from a provider who collected a cervical scraping for hrHPV testing and performed a colposcopy	1 time point	Information and instructions were available in Spanish	81% of Hispanic women reported positive feelings regarding urine self‐collection, and 71% had positive feelings about cervico‐vaginal self‐sampling^a^ 70% of Hispanic women preferred urine self‐collection, 14% preferred cervico‐vaginal self‐sampling, and 16% preferred clinician sampling^a^
Naseri et al., (2022)^39^	Stanford University Medical Center, California	Assess the concordance and acceptability of using a modified menstrual pad compared to self‐collected and clinician‐collected samples	153 106 also completed clinician collection and at‐home menstrual pad self‐collection Age range: 18 years to menopause age	14.5%	Q‐Pad (Qvin™, Menlo Park, CA), a modified menstrual pad; vaginal self‐sampling instrument was not specified	Participants were asked to self‐collect a vaginal swab (at the clinic) and self‐collect menstrual blood using the Q‐Pad (at home). A clinician‐collected cervical sample was obtained at the clinic	At initial clinic visit, self‐collected vaginal swab and clinician‐collected sample was obtained. Menstrual pads returned within 2 months	Not specified	92% stated preference for self‐collection over clinician‐collected samples^b^. Of these, 94% preferred the menstrual pad over the self‐collected vaginal swab^b^ 22.9% of participants opted out of participating in vaginal self‐sampling due to discomfort with procedure^b^
Randomized intervention studies with mixed samples									
Carrasquillo et al., (2018)^32^	South Florida	Compare the effects of three interventions (one of which included self‐sampling) on completion of cervical cancer screening (defined as either obtaining a Pap test or completing self‐sampling)	601 at baseline; 523 completed follow‐up Age range: 30–65 years	59%	Cervico‐vaginal sampling using the POI/NIH self‐sampler	Participants randomized into one of three groups: (1) brochure on cervical cancer screening and information on how to obtain a Pap test (outreach group); (2) CHW‐led education and navigation to local clinic for a Pap test (navigation group); or (3) CHW‐led education and choice of navigation to clinic for a Pap test or self‐sampling (self‐swab group). Self‐sampling was completed at the time of the education visit (location not specified)	Baseline assessment and 6‐month follow‐up.	Brochures were in the participant's preferred language and developed with input from clinicians, CHWs, and community advisory boards. Interview items were piloted and had significant input from community partners	Participants^b^ in the self‐swab group were more likely to complete screening compared to the other two conditions 64% of participants in the self‐swab group^b^ opted for self‐sampling
Kobetz et al., (2018)^33^	South Florida	Compare a mailed vs. in‐person HPV self‐sampling intervention on completion of self‐sampling	600 at baseline, 425 completed follow‐up Age range: 30–65 years	65.1%	Cervico‐vaginal sampling using the POI/NIH self‐sampler	Women were randomized into either the In‐Person Self‐Sampling (IP + SS) or the Mailed (SS + Mail) intervention arms. The IP + SS group received a 30‐min visit by a CHW and were asked to complete the self‐sampling during the visit (or later and mail their sample back). The SS + Mail arm received a self‐sampling kit by mail and was provided the same information by a CHW via a phone call. Samples collected at home and mailed back	Baseline assessment and 6‐month follow‐up	CHWs were members of the respective communities and underwent extensive training. Information was delivered in the participant's native language	81% of the IP + SS group and 71.6% of the SS + Mail intervention arms completed self‐sampling^b^

Abbreviations: CHW, Community Health Worker; IP, in‐person; POI/NIH, Preventive Oncology International/National Institute of Health; SS, self‐sampling.

^a^ Results represent the proportion of the specified Latina or Hispanic sample.

^b^ Results represent the proportion of the whole (mixed) sample.

### 3.2 Study participants and recruitment

Because national guidelines for cervical cancer screening are updated regularly, inclusion criteria for previous cytology testing varied across studies. Four studies included women who had not had a Pap test in the last 3 years,^32^, ^33^, ^37^, ^38^ two others restricted the criteria to a single year,^31^, ^34^ the remaining did not specify^35^, ^41^ or had some other criteria.^36^, ^39^, ^40^ Seven of the studies recruited participants in either a clinical setting^34^, ^35^, ^36^, ^37^, ^39^, ^40^ or community health organization^38^; the remaining four studies recruited participants from non‐clinical settings,^31^, ^32^, ^33^, ^41^ which included community organizations, churches, and participants' homes. The majority of studies^31^, ^34^, ^35^, ^37^, ^38^, ^40^, ^41^ had samples that included 74% or greater identify as Hispanic or Latina. Two studies^32^, ^33^ recruited samples that included between 59% and 65% Hispanic or Latina participants; and only two studies^36^, ^39^ reported that Hispanic participants comprised a minority of the study sample (between 14% and 29%). The age of participants ranged from 16 to 65 years.

### 3.3 Cultural and Theoretical Considerations

Culturally tailored materials can enhance participant engagement and accommodate culture‐specific values and language preferences. Six studies described how materials were adapted for the Latino population through focus groups,^34^, ^35^ pilot‐testing,^32^, ^34^, ^41^ feedback from community partners,^32^, ^33^ or with the assistance of CHWs familiar with the community.^37^ Eight studies^31^, ^33^, ^35^, ^36^, ^37^, ^38^, ^41^, ^42^ explicitly stated that study materials and information were provided in Spanish. These materials included informational brochures, presentations, and study assessments. Two other studies^34^, ^39^ did not explicitly mention language, and the remaining study^40^ was assumed to be available in Spanish since it was conducted in Puerto Rico.

Additionally, the use of theoretical frameworks allows for the comprehensive integration of cultural values throughout study materials and procedures, as opposed to mere language adaptation. However, only three articles included a description of how theoretical frameworks were used to guide study content or procedures. Carrasquillo et al.^42^ utilized Community‐Based Participatory Research (CBPR) principles. They worked closely with two community partners, one being a Federally Qualified Health Center, through all phases of the intervention. Similarly, Kobetz et al.^43^ utilized the Socio‐Ecological Model to orient their CBPR approach. Finally, the educational presentation provided to participants in Penaranda et al.^35^ was guided by selected constructs from the Health Belief Model (e.g., perceived severity).

### 3.4 Self‐sampling setting and device(s)

Five studies^31^, ^32^, ^33^, ^35^, ^37^ utilized community health workers (CHW) in study procedures. In two of these studies,^35^, ^37^ the CHW operated within a clinical setting; in the remaining three studies, CHWs conducted activities in non‐clinical settings (e.g., participant's home).^31^, ^32^, ^33^

Seven studies involved self‐collection of samples at a clinic^34^, ^35^, ^36^, ^37^, ^39^, ^40^ or community health location,^38^ and four studies were conducted in the participant's home^41^ or utilized an agreed‐upon community location (which could be the participant's home).^31^, ^32^, ^33^ Only two of the studies^33^, ^39^ asked participants to mail samples back; in all of the other studies, participants returned their self‐collected sample(s) in person.

All of the studies included a device for cervico‐vaginal self‐collection, such as a swab or brush that can be inserted into the vaginal canal. In addition, one study also collected menstrual blood using modified pads,^39^ and several studies collected anal samples,^40^ urine samples,^36^ and oral and anal samples.^41^ In five of the studies, a clinician‐collected sample was also obtained.^31^, ^34^, ^36^, ^39^, ^40^

### 3.5 Self‐sampling acceptability

The studies used varied measures to assess women's acceptability of self‐sampling. Two studies assessed the acceptability of HPV self‐sampling only, with no comparison to physician sampling or other forms of sampling.^37^, ^38^ In these studies, Latina participants expressed very favorable opinions of self‐sampling, with 83%–99% reporting that self‐sampling was easy to perform. Further, when asked to state a preference for self‐sampling or a clinician‐performed Pap test, 89% of Latina participants indicated that they would choose self‐sampling over a clinician‐administered Pap test in the future.^37^ Women reported that ease of use, convenience, and privacy were major factors contributing to high acceptability of self‐sampling.^37^, ^38^

Three studies assessed women who underwent both self‐sampling and had a Pap test performed by a clinician.^31^, ^34^, ^35^ One of the three studies was comprised entirely of Latina participants, and the majority (81.8%) rated the ease of use and convenience of self‐sampling over clinician sampling as excellent or very good.^31^ The other two studies had mixed study samples comprised of at least 76% Latina participants^34^, ^35^; in these studies, the majority of participants (which included some non‐Latina women) reported that self‐sampling was easy to perform (100%^35^ and 69%^34^). Among women residing along the U.S.‐Mexico border (of which 87% were Latina), acceptability of self‐sampling was rated higher than for a clinician‐administered Pap test, and all participants reported that self‐sampling was easy and convenient. When asked to express a preference, 30% stated a preference for self‐sampling, 26% preferred a clinician‐administered Pap test, and 42.7% expressed no preference.^35^ The third study reported that only 28% of Latina participants preferred self‐sampling to clinician‐collected sampling procedures,^34^ due to concerns that self‐sampling is less sensitive or accurate than a clinician‐collected sample, even though self‐sampling was acceptable to the majority of women. The findings from this study may also differ from the other published studies because all study participants were recruited from either a clinic for sexually transmitted diseases (STDs) or from a cancer screening clinic and had been screened for cervical cancer within the past 3 years. Further, the authors noted that preferences for self‐sampling differed across clinic sites, with women presenting at a cancer screening clinic expressing greater desire for a clinician‐administered test and lower preferences for a home‐based option that did not require “coming in for an appointment” compared to women who were recruited from the STD clinic.^34^ Thus, among screening‐adherent women who are motivated to seek care specifically at a cancer screening clinic, the option to self‐collect one's own sample may be perceived to be less accurate or more likely to potentially ‘miss’ a cancer compared to a clinician‐administered test.

Four studies compared women's preferences across sampling sites (cervico‐vaginal, anal, oral) and sample types (cervico‐vaginal swab or broom, urine, menstrual blood).^36^, ^39^, ^40^, ^41^ Two of these studies were conducted in Puerto Rico and had study samples that included over 88% Latina participants. In these studies, women preferred self‐collection of vaginal samples to physician‐collected sampling.^40^, ^41^ However, collection of oral rinse samples was preferred to vaginal sampling.^41^ The two remaining studies also reported preferences for self‐sampling over clinician‐sampling,^36^, ^39^ although these studies included only a minority of Latina or Hispanic participants. However, subgroup analyses of Hispanic women only revealed that the majority reported positive feelings toward urine self‐collection (81%) and cervico‐vaginal self‐sampling (71%).^36^

Only two studies utilized a randomized trial design, and both of these were conducted in South Florida with over 59% Latina participants (the remainder was comprised of Haitian and non‐Hispanic Black participants). Results were reported across the entire sample. In brief, a greater proportion of women provided with a self‐sampling option completed cervical cancer screening (77%) compared to women randomized to outreach only (which included a brochure about screening and information on where to obtain a Pap test; 31%) or to CHW‐led education and navigation to a clinic for a Pap test (43%).^32^ A subsequent randomized trial evaluated whether delivering self‐sampling kits in‐person was superior to a mailed kit.^33^ Completion of self‐sampling was slightly higher when delivered in‐person by a CHW (81%) compared to delivered via US mail (71.6%).^33^ Acceptability was not directly measured in either study, but it can be inferred from the high completion rates that women were comfortable with self‐sampling and deemed it to be an acceptable option.

## 4 DISCUSSION

This is the first systematic review of the acceptability of HPV self‐sampling among U.S. Latina women. Despite the different methodologies used across studies, our findings indicate that acceptability of self‐sampling is high in this population, with women reporting that self‐sampling is easy to perform and convenient, offers greater privacy, and is less embarrassing to undergo than a clinical exam. The primary disadvantage of self‐sampling that was reported was the fear that one was not collecting the sample correctly.^34^ Clinician‐collected samples were perceived to be more accurate amid this concern. Finally, although cervico‐vaginal self‐sampling was acceptable to women, the collection of other samples (e.g., oral rinse, urine, menstrual blood) was preferred to vaginal sampling.^36^, ^39^, ^41^

Our findings are consistent with those reported in other populations. A systematic review of studies conducted across 24 countries also indicated high acceptability of self‐sampling, with women reporting a greater preference for self‐sampling over clinician sampling due to its ease and convenience.^44^ A more recent review of women's values and preferences around self‐sampling also revealed key preferences for setting, with the ability to collect one's sample at home being preferable to self‐collecting one's sample at a clinic due to these same reasons (ease, convenience, and privacy).^45^ In the present review, we did not observe any distinct patterns in preference for self‐sampling by setting (clinic vs. home); however, the location of self‐collection was not always specified or it was variable even within a particular study (e.g., self‐sampling could be performed at the participant's home or other convenient location).

Since most studies had the potential to be completed within a single interaction with research or healthcare staff, there were limited opportunities to provide more comprehensive education and information about cervical cancer risk and the benefits of screening and early detection, which is a potential disadvantage of this approach. Indeed, delivery of educational content was not a primary focus in many of the studies. If offered, the educational information provided was brief and did not allow for repetition of the material in multiple instances. In studies where educational information was provided, it was delivered by CHWs and/or with Spanish‐language materials, which underscores the need to utilize culturally‐ and linguistically‐appropriate methods for engaging this population. Although it is acknowledged that the addition of CHWs or health education content could lead to greater burden (i.e., longer visits), recent studies offer some potential strategies to consider.^46^ For example, 52% of medically underserved women (including Latina women) reported that they would be willing to participate in social‐media interventions about HPV self‐testing.^46^ Similarly, a recent systematic review reported that video interventions can be effective in increasing cervical cancer screening broadly.^47^ Further, video interventions that were culturally tailored were more effective in promoting screening uptake compared to those that provided information only.^47^

At present, the cost‐effectiveness of implementing a broad self‐sampling program for cervical cancer screening in this population is unclear. Additional research is needed to assess the cost–benefit ratio for delivering such programs over the long‐term. Future studies should also prioritize rigorous follow‐up with participants. Given that some women may not be eligible to participate in available government‐funded programs due to immigration status or other factors, finding sufficient support for follow‐up care remains a persistent challenge.^48^ This may necessitate setting aside additional resources to help underserved women obtain medical and financial assistance for diagnostic healthcare services.^49^, ^50^ It may also require expanding the eligibility criteria for state and/or federally‐funded programs that provide low‐cost cancer screening and treatment services for underserved women.

Finally, it is acknowledged that offering a self‐sampling option for cervical cancer screening is not intended to replace attendance at routine care visits with one's healthcare provider. Rather, the goal is to promote women's active engagement in their preventive care and to increase acceptance of screening. Qualitative data in other samples suggest that self‐sampling may help achieve these goals, especially since some women have reported feeling less embarrassed about screening following the performance of self‐collection.^51^ Other indirect benefits that may result from self‐sampling include increasing women's comfort and autonomy with their bodies, emphasizing the importance of screening for maintaining good health, and reducing embarrassment associated with gynecologic exams in general.^51^, ^52^

Strengths of this review include the use of multiple databases to identify a comprehensive list of articles and the inclusion of studies published in Spanish or English. However, some limitations should be noted. First, nine studies included women who had been screened in the past 3 years for cervical cancer, were recruited at or through a clinic, or had previously tested positive for HPV—indicating a potential sampling bias. Since these participants already had access to healthcare and/or demonstrated health‐seeking behaviors, they may not be representative of unscreened or underscreened women. Thus, the acceptability of self‐sampling may be higher in these samples that have healthcare access or experience with cancer‐screening behaviors. Second, most studies were single‐arm studies (in which all participants underwent all study procedures); only two studies utilized a randomized trial design. Therefore, it is difficult to evaluate whether uptake of self‐sampling would vary across conditions or settings. Third, most studies were descriptive in nature and had modest sample sizes or reported overall findings across a mixed sample of both Latina and non‐Latina participants, which limits the extent to which we can draw definitive conclusions about the acceptability of self‐sampling approaches in the US Latina population. The ability to disaggregate data across Hispanic/Latino subpopulations is also critically needed in future studies as various factors (e.g., immigration status, English language proficiency) that differ across subgroups likely also contribute to women's willingness to participate in self‐sampling and cervical cancer screening.^53^ Finally, the concentration of studies in Florida, California, and Puerto Rico highlights specific regional initiatives tailored to the needs of Latinas in those areas; but given the large population of Latina residents across Texas, New York, and Arizona,^54^ it may be fruitful to expand studies across a broader region. Overall, however, the findings from this systematic review suggest that self‐sampling approaches for cervical cancer screening may offer a promising option to consider for future clinical research involving this population.

## 5 CONCLUSION

Providing alternatives to clinic‐based cervical cancer screening are acceptable and could enhance screening participation and engagement among underscreened US Latinas facing barriers to traditional screening. HPV self‐sampling has emerged as a promising approach to address cervical cancer disparities by introducing evidence‐based strategies for HPV DNA testing and increasing screening accessibility. This holds particular significance as Latinas encounter distinct barriers to care including language obstacles, difficulties in accessing care, and experiences of racism and discrimination. Findings from this systematic review offer guidance for future clinic‐ and community‐based programs aimed at increasing participation in recommended cervical cancer screening, with the ultimate goal of eliminating disparities in cervical cancer and enhancing women's health.

## AUTHOR CONTRIBUTIONS

**Marisol S. Cora‐Cruz:** Conceptualization (lead); data curation (lead); formal analysis (lead); investigation (lead); project administration (equal); supervision (equal); writing – original draft (lead); writing – review and editing (equal). **Omar Martinez:** Conceptualization (equal); formal analysis (supporting); funding acquisition (equal); investigation (equal); methodology (lead); project administration (equal); resources (equal); supervision (equal); writing – review and editing (supporting). **Sophia Perez:** Data curation (supporting); formal analysis (supporting); investigation (supporting); writing – review and editing (supporting). **Carolyn Y. Fang:** Conceptualization (equal); funding acquisition (lead); investigation (supporting); project administration (equal); resources (supporting); supervision (equal); writing – original draft (supporting); writing – review and editing (supporting).

## CONFLICT OF INTEREST STATEMENT

The authors declare no conflicts of interest.

## ETHICS STATEMENT

N/A—No human subjects. This manuscript reports the findings from a systematic review of the literature.

## PRECIS

This systematic review summarizes the acceptability of HPV self‐sampling for cervical cancer screening among US Latinas. The findings indicate that self‐sampling was deemed to be acceptable due to its ease of use, comfort, privacy and convenience. Therefore, it presents a viable option for enhancing participation in cervical cancer screening in this population.

## Supporting information


Table S1.

## Data Availability

No new data were obtained in this manuscript. This systematic review synthesizes data from existing studies. Each of the studies reviewed is cited and is publically available. Table 1 in the manuscript describes the information that was extracted from each study.
