# Supplementary material for: Evaluating human papillomavirus (HPV) self‐sampling among Latinas in the United States: A systematic review
Source: Cancer Med. 2024 Aug 16;13(16):e70098. doi: 10.1002/cam4.70098 (PMC11327612; doi:10.1002/cam4.70098)
Supplement: Supplementary file 1 — Table S1. [file CAM4-13-e70098-s001.docx]

**Supporting Table 1.** Search terms used in the PubMed and EBSCOhost database searches.

| **#** | **Key Concepts** | **PubMed** | **EBSCOhost Research Database** |
| --- | --- | --- | --- |
| **1** | Cancer | ((HPV) OR ("Alphapapillomavirus"[Mesh]) OR "Papillomaviridae"[Mesh]) AND ("Uterine Cervical Neoplasms"[Mesh] OR "cervical cancer") | HPV OR (“Papillomaviridae"[Mesh]) AND ("Uterine Cervical Neoplasms"[Mesh] OR "cervical cancer") |
| **2** | Self-Sampling | (self-sampl* OR self-collect*) | self-sampl* OR self-collect* |
| **3** | Hispanic | ((hispanic*[tiab] OR "hispanic american*"[tiab] OR "hispano*"[tiab] OR "latine*"[tiab]OR "latina*"[tiab] OR "latin"[tiab] OR "latinu*"[tiab] OR "latino"[tiab] OR "latinx*"[tiab]OR "latin american*"[tiab] OR "latin america"[Mesh] OR "spanish speak*"[tiab] OR"mexico*"[tiab] OR "Mexico"[Mesh] OR "cuban*"[tiab] OR "peruvian*"[tiab] OR"dominican*"[tiab] OR "brazilian*"[tiab] OR "central american*"[tiab] OR "costa rican*"[tiab] OR "guatemalan*"[tiab] OR "honduran*"[tiab] OR "uruguayan*"[tiab] OR"argentina"[Mesh] OR "argentine"[tiab] OR "argentinian"[tiab] OR "argentinean"[tiab]OR "panamanian*"[tiab] OR "salvadorean*"[tiab] OR "salvadoran*"[tiab] OR"salvadorian*"[tiab] OR "nicaraguan*"[tiab] OR "south america*"[tiab] OR "bolivian*"[tiab] OR "chilean*"[tiab] OR "Chile"[Mesh] OR "colombian*"[tiab] OR "ecuadorian*"[tiab] OR "paraguay*"[tiab] OR "Paraguay"[Mesh] OR "venezuelan*"[tiab] OR "puertorican*"[tiab] OR "puerto rico*"[tiab] OR "Puerto Rico"[Mesh] OR "spanish america*"[tiab] OR "boricua*"[tiab] OR "chicana*"[tiab] OR "chicano"[tiab] OR "Hispanic or Latino"[Mesh] OR "latinoamerican*"[tiab] OR "Mexican Americans"[Mesh] OR"spanish caribbean*"[tiab] OR "mexican american*"[tiab])) | hispanic OR hispanic american* OR hispano* OR latine OR latina* OR latin OR latinu* OR latino OR latinx*OR latin america* OR spanish speak* OR mexico* OR Mexico[Mesh] OR cuban* OR peruvian* OR dominican* OR brazilian* OR central american* OR costa rican* OR guatemalan* OR honduran* OR uruguayan* OR argentina[Mesh] OR argentine OR argentinian OR argentinean OR panamanian* OR salvadorean* OR salvadoran* OR salvadorian* OR nicaraguan* OR south america* OR bolivian* OR chilean* OR Chile[Mesh] OR colombian* OR ecuadorian* OR paraguay* OR Paraguay[Mesh] OR venezuelan* OR puertorican* OR puerto rico* OR Puerto Rico[Mesh] OR spanish america* OR boricua* OR chicana* OR chicano OR Hispanic OR Latino[Mesh] OR latinoamerican* OR Mexican Americans[Mesh] OR spanish caribbean* OR mexican american* |
| **4** | Programs/Interventions | program OR intervention | program OR intervention |
|  | **Additional Information** | Concept Building Query Search: #1 AND #2 AND #3 AND #4 | Utilized AND feature to connect all searches. The database itself removed duplicates once clicked on the second page. All journals were selected. Range: 2003-2023. |
